# Effects of high-intensity interval training in a cold environment on arterial stiffness and cerebral hemodynamics in sedentary Chinese college female students post-COVID-19

**DOI:** 10.3389/fneur.2024.1466549

**Published:** 2024-11-05

**Authors:** Xiangyuan Chen, Niyuan Hu, Huifeng Han, Guoliang Cai, Ying Qin

**Affiliations:** College of Sports and Human Sciences, Harbin Sport University, Harbin, China

**Keywords:** high-intensity interval training, arterial stiffness, cold environment, cardiovascular health, public health

## Abstract

Many patients with COVID-19 experience increased arterial stiffness and abnormal cerebral hemodynamics. Although previous studies have explored the effects of cold environments on cardiovascular health and cerebral hemodynamics, there is still no research on the changes in cardiovascular and cerebral hemodynamics in sedentary female students recovering from COVID-19 while performing high-intensity interval training (HIIT) in cold environments. This study investigates the effects of 1 week of HIIT in a cold environment on cerebral hemodynamics and arterial stiffness (AS) in sedentary female college students, providing new insights into the pathophysiological mechanisms in this specific context. Thirty-six participants were randomly divided into a control group (*n* = 12), a room temperature (RE) group (*n* = 12), and a cold environment (CE) group (*n* = 12). HIIT was performed for four 4-min running training sessions, with a 4-min interval between each training session, The training duration was 1 week, with a frequency of 2 sessions per day, while the control group did not undergo any training. After training, the AS in the CE group significantly decreased (*p* < 0.05), with an average reduction of 11% in brachial-ankle pulse wave velocity, showing a significantly greater improvement compared to the RE group and the control group (*p* < 0.05), while no significant changes were observed in the RE group (*p* > 0.05). In the Y-Balance Tests (YBTs), the concentrations of cerebral oxygenated hemoglobin and total hemoglobin significantly increased (*p* < 0.05) during unilateral leg support tests in both the CE and RE groups, and the increase of CE group is greater than that of RE group. In contrast, in the control group, the concentrations of cerebral oxygenated hemoglobin and total hemoglobin significantly decreased during left leg support (*p* < 0.05). Our study found that performing HIIT in a cold environment not only effectively reduces AS in sedentary female college students after COVID-19, improves cardiovascular function, but also significantly enhances cerebral hemodynamics, helping them alleviate the negative impacts of post-COVID-19 sequelae and sedentary behavior on health. Future research should further explore the mechanisms by which sedentary behavior, post-COVID-19 recovery status, and adaptation to cold environments collectively influence cardiovascular function and cerebral hemodynamics, providing a more comprehensive understanding of these factors.

## 1 Introduction

COVID-19 (Coronavirus Disease 2019) is an infectious disease caused by the novel coronavirus SARS-CoV-2. During the infection, the virus can lead to pathological changes in various organs. Even after recovering from COVID-19, many patients continue to experience a range of sequelae (1). For example, decreased immunity, metabolic and endocrine disorders, cognitive impairment, cardiovascular dysfunction, and increased all-cause mortality can occur (2–4). The association between these non-communicable diseases and sedentary behavior has also been well established (5), and sedentary behavior habits may aggravate the development of COVID-19 sequelae (6).

After infection, COVID-19 patients develop systemic inflammation and release cytokines, which damage the integrity of the vascular endothelium. The cytokine cascade reduces the bioavailability of nitric oxide (NO), thereby increasing arterial stiffness (AS) (7, 8). The Windkessel effect refers to the phenomenon where arteries, particularly large arteries (such as the aorta), buffer the pressure fluctuations generated by the ejection of blood into the vascular system during the heart's contraction and relaxation phases through their elastic properties, thereby maintaining continuous and steady blood flow and blood pressure (9). Increased AS can weaken the Windkessel effect, resulting in increased pulse pressure in the aorta and large blood vessels (10). Such changes impact microvessels in some high-flow, low-resistance organs (such as the brain, kidneys, retina, and heart) (11). Increased blood flow pressure in the brain can cause changes in cerebral microcirculation (such as vascular wall hypertrophy and arteriolar rarefaction), leading to chronic cerebral ischemia, white matter hyperintensities, and clinically unrecognized small focal cerebral infarctions (12). Studies have shown that even patients with mild COVID-19 infection may experience increased AS, brain structural changes, and cognitive impairment (13). Therefore, effective rehabilitation strategies are crucial for promoting recovery from COVID-19 sequelae and reducing the harm caused by sedentary behavior.

Previous studies have shown that appropriate physical exercise, particularly high-intensity interval training (HIIT), can effectively improve AS and cerebral hemodynamics (14–16). The reason for selecting HIIT in this study is that the training approach, which involves short bursts of high-intensity exercise interspersed with periods of low-intensity activity or rest, not only allows for the accumulation of multiple high-intensity exercise bouts within a shorter period but also provides a greater stimulus to the body, leading to enhanced exceeding compensation (17). Additionally, HIIT exerts greater shear stress on the vascular walls, inducing more significant hemodynamic changes and notably increasing nitric oxide production and utilization in both the vascular system and the brain (18, 19). These effects are crucial for reducing AS, enhancing cerebral blood flow perfusion, promoting microvascular remodeling in the brain, improving cortical metabolic capacity, strengthening the blood-brain barrier, and enhancing brain plasticity (20).

During exercise, adding a cold environment to increase exercise stimulation can mediate changes in arterial diameter or greater blood flow and shear stress, thereby enhancing vascular adaptation to chronic exercise training. Existing research suggests that when the temperature drops below 11°C, if an individual cannot generate sufficient heat to counteract the cold, it may lead to a decrease in the temperature of peripheral nerves and muscles (21). This can result in excessive muscle contraction or stiffness, excessive constriction of peripheral blood vessels, and excessive depletion of glycogen, ultimately negatively impacting physical function and exercise performance (22). However, at a temperature of 14°C, moderate cold exposure may enhance venous blood return by increasing hydrostatic pressure within blood vessels through augmented peripheral vasoconstriction (23–25). Additionally, this temperature can improve endurance performance by helping participants maintain a better physical and mental state during training (26). At the same time, some substances have a unique “temperature-dependent” release pattern. For example, when exercising in a cold environment, the concentrations of lactic acid, irisin, fibroblast growth factor 21 (FGF21), and myristic acid are significantly increased (27–29). Lactic acid can provide energy for brain activity and can also regulate brain function as a signaling molecule in combination with irisin, FGF21, and myristic acid (30).

Cold exposure mediates cardiovascular responses, including sympathetic excitation-induced cutaneous capillary constriction and increased blood pressure. Cutaneous capillary constriction is the main mechanism for limiting heat loss and maintaining core temperature. Studies have shown that an environment at 14°C is conducive to parasympathetic activation in females, which can effectively prevent the adverse effects of sympathetic overactivation and promote synergistic effects between the sympathetic and parasympathetic nerves, ensuring the stability of the cardiovascular system (24, 31). In the cerebrovascular system, unlike in the peripheral and microvascular systems, cold exposure causes a decrease in vascular resistance, leading to an increase in cerebral blood flow velocity (CBFv). Increased facial cooling during exercise promotes exercise-induced increases in the CBFv (32, 33). Exercise in a cold environment may lead to a greater increase in the CBFv than exercise in a normal temperature environment, which may translate to improved cerebrovascular system function.

Based on the above discussion, we believe that incorporating cold exposure during exercise could be a potential strategy to enhance or maintain cardiovascular and cerebrovascular function in young, healthy individuals (33). While the effects of HIIT on cardiovascular health and cerebral hemodynamics have been extensively studied, the specific impact of cold environments on AS in post-COVID-19 patients has not been fully explored. Therefore, we investigated the effects of a 1-week HIIT program in a cold environment on cerebral hemodynamics and AS in sedentary female college students. We hypothesize that, compared to HIIT in a normal temperature environment, HIIT in a cold environment may result in more significant improvements in cerebral hemodynamics and AS in sedentary female college students.

## 2 Materials and methods

### 2.1 Participants

This study involved the recruitment of 36 sedentary female college students who met the inclusion criteria after COVID-19 [cold environment (CE) group: *N* = 12; room temperature (RE) group: *N* = 12; control group: *N* = 12]. The inclusion criteria were patients aged between 19 and 26 years, patients with a right dominant leg, patients who had 2 or more mild to moderate COVID-19 symptoms, and sedentary female college students with low physical activity according to the International Physical Activity Questionnaire (IPAQ). To ensure the reliability of the experimental results, this study assessed participants' tolerance to different temperatures through a questionnaire survey and selected those who were suitable for the temperature settings of this experiment. The exclusion criteria were a history of cardiovascular disease (CVD), metabolic diseases, severe respiratory diseases, and musculoskeletal diseases that hindered the participants' physical activity. This study was approved by the Human Experiment Ethics Committee of Harbin Institute of Physical Education (Approval No. 202314). All volunteers signed written informed consent in accordance with the Declaration of Helsinki.

### 2.2 Experimental procedure

Three days before the formal experiment, participants wore loose and comfortable sportswear to the laboratory for baseline assessment (pretest), and the simplified version of the International Physical Activity Questionnaire was used to screen for sedentary time and physical activity. Afterward, under the guidance and supervision of two professional coaches, the cold environment group and the room temperature group were treated twice a day between 9–11 am and 14–16 pm for a period of 1 week, while the control group, maintaining their sedentary lifestyle, remained in the same temperature environment as the RE without any interventions. The training results were repeatedly measured in the morning of the next 3 days after the last training (posttest). To minimize the influence of biological rhythms and dietary factors, the food consumed by the participants during the week was uniformly rationed within the prescribed time period of the day. Participants were required to fast for 12 h before the two measurements (pretest and posttest) at a room temperature of 26°C, as cardiovascular function is relatively stable at this temperature, with lower blood pressure and heart rate variability (34), and not to eat after 20:00 the previous day or 4 h before the measurement. The test time was uniformly between 9 and 12 am. Participants were required to go to bed before 23:00 the day before the test to ensure adequate rest. In the pre- and post-intervention evaluations, to prevent measurement errors, all the results were measured by the same evaluator in the same laboratory, and the intervention process was completed at the training center of the Harbin Institute of Physical Education.

**Figure 1:**
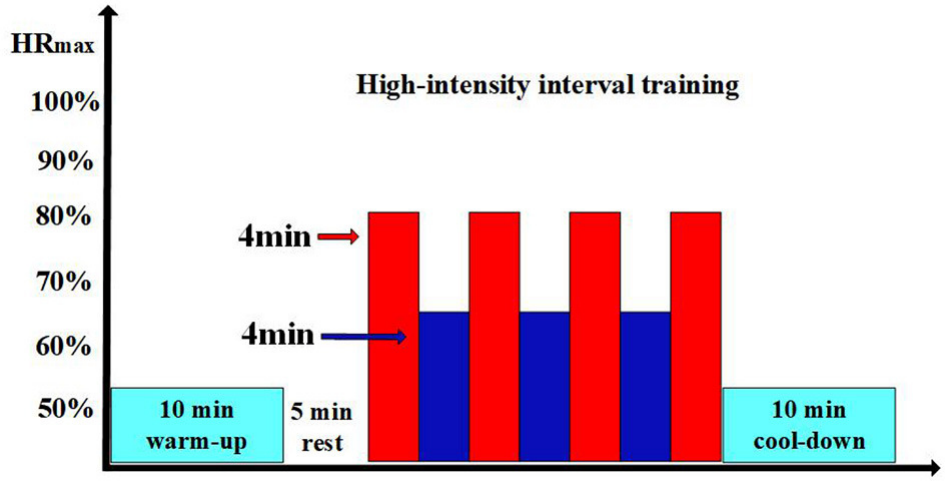
Experimental process. The light blue sections represent the 10-min warm-up or 10-min cool-down; the blank sections represent the 5-min rest periods; in the HIIT training, the red sections indicate the high-intensity exercise phases; and the dark blue sections represent the low-intensity recovery phases.

The temperature for both the control group and the room temperature group was set at 22°C, the cold environment group was set at 14°C, with a relative humidity (RH) of 55% for all three groups (33, 35). These temperature differences are intended to effectively contrast the environmental effects on cardiovascular function. According to the American College of Sports Medicine (ACSM), the temperature for all physical activity areas should be maintained between 20°C and 22.2°C, with relative humidity (RH) below 60% (36). Previous studies have found that 22°C may be a comfortable temperature for the human body and is an ideal choice for exercise, as it promotes stable energy metabolism which avoids placing an adverse burden on the cardiovascular system due to metabolic factors, and helps enhance participants' motivation for training and work (37–39). The control group maintained a sedentary lifestyle for at least 6 h daily in a 22°C environment. Participants were required to wear comfortable shorts, short-sleeve t-shirts, and sneakers for training or sedentary activities. Both the room temperature and cold environment groups performed a 10-min warm-up before each training session in 22°C environment. Both groups performed low-intensity (50%−60% of peak heart rate) exercise. After a thorough warm-up, the formal training session began after a 5-min rest. The HIIT program consisted of four 4-min brisk sprints on a treadmill (75%−80% of peak heart rate and a Borg Rating of Perceived Exertion of 15–18), with a 4-min active recovery period (60%−70% of peak heart rate) between each session until the end of the training, as shown in Figure 1. The control group maintained a sedentary habit of 6 h or more per day. Target intensity was checked every 2 min during the exercise, and maximum heart rate was calculated using the formula 220 minus age. After the training session, a 10-min cool-down session was performed. Heart rate was monitored using a heart rate monitor (Polar Electro, Kempele, Finland). The speed and incline of the treadmill were adjusted according to individual conditions to ensure that each training session was performed at the prescribed heart rate. After the afternoon HIIT training, we used the 6–20 RPE scale to assess participants' feelings throughout the day of HIIT training. This approach aims to provide a comprehensive understanding of participants' subjective fatigue and effort levels during the day's training. Subsequently, we will continue to monitor and record participants' recovery and training effects to evaluate and analyze them the following day. This method allows us to assess the long-term impact of HIIT training on participants and adjust future training plans to optimize results and reduce potential fatigue or overtraining risks.

### 2.3 Measurement

#### 2.3.1 Resting heart rate, blood pressure, and arterial stiffness

AS is measured by simultaneous measurement of baPWV, resting heart rate (HR), systolic blood pressure (SBP), and diastolic blood pressure (DBP) using a non-invasive blood vessel testing device (BP-203RPEIII, Omron Medical Technology, Japan). Subjects were required to fast for 4 h prior to the measurements and to abstain from consuming caffeinated beverages such as coffee or tea for 6 h before the measurement. Before testing, participants rested in a supine position on a testing bed for at least 5 min to allow their heart rate and blood pressure to stabilize. Following this, trained professionals placed pneumatic pressure cuffs on both sides of the participants' ankles (2–3 cm above the ankle joint) and both upper arms (approximately 2–3 cm above the antecubital fossa). baPWV was automatically calculated as the arterial segment length (from the brachial artery to the ankle, automatically calculated based on height) divided by the pulse wave propagation time.

#### 2.3.2 Near-infrared functional neuroradiography data

**Figure 2:**
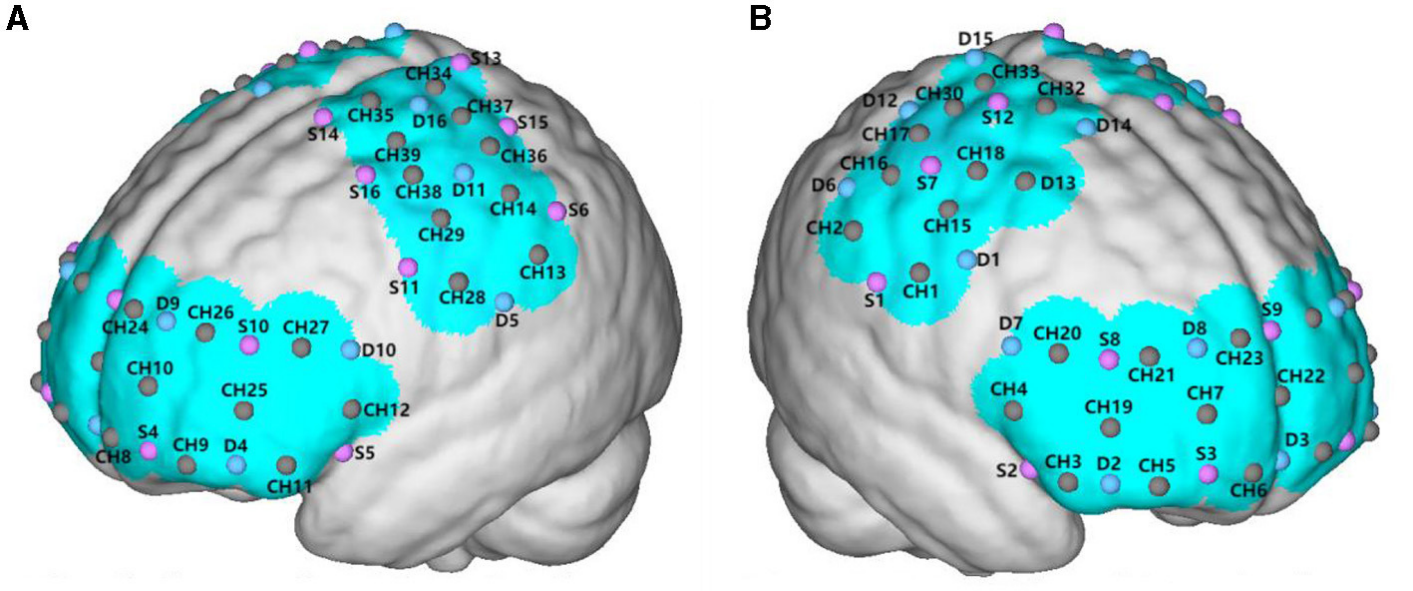
The red dots represent the locations where the detectors are placed; the blue dots represent the locations of the light sources; and the brown dots represent the channels formed between the light sources and detectors. **(A)** The left cerebral hemisphere. **(B)** The right cerebral hemisphere.

fNIRS data were recorded using a multichannel portable Nirsmart (NirScan-3000 C, Danyang Huichuang Medical Equipment Co., Ltd., China). The system uses two wavelengths (730 nm and 850 nm) with a sampling frequency of 11 Hz. The head cap for this experiment was designed based on the 10/20 international standard lead system. The head cap was placed on the participant's head, referenced to the Cz position, and visually aligned in the sagittal plane. The probe for each subject included 16 detectors (probes) and 16 emitters (light sources), forming 39 channels. The distance between the detectors and emitters was 30 mm. It can effectively cover and collect changes in oxygenated hemoglobin (HBO) signals and total hemoglobin (HBT) concentrations in the frontal cortex area. The HBT is a marker of cerebral blood volume and can well reflect changes in cerebral hemodynamics. HBO can reflect the activation of the brain during tasks (40–42). We divided the measured brain area into 6 regions of interest (ROIs), including the premotor cortex (PMC), supplementary motor cortex (SMC), primary motor cortex (M1), primary sensory cortex (S1), frontopolar area (FPA), and dorsolateral prefrontal cortex (DLPFC). The PMC and SMC are close to each other, so they were monitored together. The PMC and SMC left channels were CH28, CH29, CH35, CH38, and CH39, PMC and SMC right channels were CH1, CH15, CH18, CH31, and CH32. The FPA left side included CH8, CH9, CH10, CH24, and CH26, and FPA right side included CH5, CH6, CH7, CH21, and CH23. The M1, left side included CH34, CH36, and CH37, and M1 right side included CH17, CH33, and CH30. The S1 left side included CH13 and CH14, and S1 right side included CH2 and CH16. The DLPFC left side included CH12, CH25, and CH27 and DLPFC right side included CH4, CH19, and CH20, as shown in Figure 2. The fNIRS data analysis methods can be found in Appendix 1.

#### 2.3.3 Y balance test

The Y-balance tests (YBTs) was used to assess dynamic postural stability. The participants practiced the test three times each in the anterior (A), posteromedial (PM), and posterolateral (PL) directions before the test. Participants stood barefoot on one leg to eliminate the additional balance support provided by shoes. During the test, they placed their big toe on the center of the grid-marked test area. They then placed their hand on their pelvis and, while maintaining stability on their right leg, pushed the “reach indicator” with their left leg as far as possible anteriorly, posterolaterally, and posteromedially. They pushed as far as possible in each direction and then returned their left leg to the starting position. The maximum reach distance was recorded to the nearest 0.5 cm. The test failed and was repeated (1) if the extended foot touched the ground, (2) if the participant's center of gravity shifted to the extended leg, (3) if the extended foot was not returned to the starting position, or (4) if the hand was not kept on the pelvis. Three successful trials were collected for each direction. Leg length was recorded by measuring the distance between the anterior superior iliac spine and the medial malleolus. The composite score for the YBTs was calculated by averaging the scores in each direction and normalizing for the participant's individual leg length. Leg length was measured from the anterior superior iliac spine to the medial malleolus.


$$\begin{array}{l} YBTs\;comprehensive\;score\;=\;[(A\;+\;PM\;+\;PL)/\\ \;(leg\;length\;\times \;3)]\;\times \;100\% \end{array}$$

#### 2.3.4 Body composition

Body composition was measured using the multi-frequency bio-impedance method. Before measurement, the skin of the participants' hands and feet and the electrodes were cleaned with alcohol. After drying, the participants were asked to stand barefoot and hold the handle of the analyzer in an upright position for measurement.

### 2.4 Statistical analysis

SPSS 22.0 was used for data analysis, and GraphPad Prism 9 was used for data visualization. The experimental data are expressed as the mean ± standard deviation (mean ± SD). To analyze the body weight, body fat percentage, BMI, muscle mass, YBTs score, AS, pulse pressure, systolic blood pressure, diastolic blood pressure, HBO concentration and HBT concentration in the frontal cortex area, the initial basic data were classified and tested for a normal distribution, and a paired sample *t* test was subsequently used to analyze the changes before and after in the group. For data that did not conform to a normal distribution, non-parametric tests were used to calculate the difference. One-way variance tests were used to analyze the differences between the groups, and *P* ≤ 0.05 was considered to indicate statistical significance. In general, this study used rigorous statistical methods to analyze the data to ensure the reliability of the results.

## 3 Results

Table 1 summarizes the characteristics of all participants. The results of the variance homogeneity test showed that there were no significant differences in age, height, weight, BMI, body fat percentage, muscle mass, sedentary time, or physical activity level among the three groups (*p* > 0.05). No adverse events occurred during the intervention period.

**Table 1:** Characteristics of participants.

**Group**	**Control (*n =* 12)**	**RE (*n =* 12)**	**CE (*n =* 12)**	***P*-value**
Age (years)	23.75 ± 0.75	24.0 ± 1.04	23.6 ± 0.9	
Hight (cm)	167.54 ± 6.18	172.58 ± 7.65	171.44 ± 6.65	
Body weight (kg)	64.15 ± 8.72	66.0 ± 9.15	65.87 ± 9.97	0.863
BMI (kg/m^2^)	22.68 ± 2.33	22.09 ± 2.09	22.33 ± 2.62	0.827
BF%	22.32 ± 4.43	22.29 ± 4.91	22.66 ± 7.02	0.982
SLM	45.66 ± 8.3	48.24 ± 7.77	47.78 ± 7.4	0.693
Sedentary time (hour/day)	8.79 ± 1.34	8.21 ± 1.54	8.46 ± 1.49	0.621
Physical activity (MET-min/week)	697.17 ± 216.84	682.92 ± 199.32	690.92 ± 191.84	0.985

The data are expressed as the mean ± standard deviation (SD). BMI, body mass index; BF%, body fat percentage; SLM, soft lean mass.

### 3.1 Changes in body composition, blood pressure, heart rate, and YBTs of participants before and after training

As shown in Table 2, after the intervention, the body weight (T = 2.596, *P* = 0.025), body fat percentage (T = 2.479, *P* = 0.031), and SBP (T = 2.771, *P* = 0.018) of the cold environment group were significantly lower (*p* < 0.05), and the YBTs score (T = −5.353, *P* < 0.001) was significantly greater than that of the pretest group (*p* < 0.05). Compared with those of the pretest group, the body fat percentage (T = 4.850, *P* < 0.001) and SBP (T = 2.666, *P* = 0.022) of the room temperature group were significantly lower (*p* < 0.05), and the YBTs score (T = −2.337, *P* = 0.039) was significantly greater (*p* < 0.05). The heart rate (T = 3.836, *P* = 0.003), SBP (T = −2.537, *P* = 0.028), and PP (T = −2.275, *P* = 0.044) of the control group were significantly greater than those before the test (*p* < 0.05). Intergroup comparisons of the three groups revealed that the SBP (F = 3.459, *P* = 0.043) and PP (F = 6.129, *P* = 0.005) was significantly different after training (*p* < 0.05). The PP in the control group was significantly higher than that in the CE and RE. Additionally, the post-test mean SBP in the CE and RE was significantly lower than that in the control group.

**Table 2:** Body composition, blood pressure, heart rate, and YBTs scores of participants before and after training (mean ± SD).

	**Control (*****n*** **=** **12)**		**RE (*****n*** **=** **12)**		**CE (*****n*** **=** **12)**		**F**		***P*** **-value**	
	**Pre**	**Post**	**Pre**	**Post**	**Pre**	**Post**	**Pre**	**Post**	**Pre**	**Post**
Weight (kg)	64.15 ± 8.72	63.93 ± 9.09	66.0 ± 9.15	64.98 ± 9.6	65.87 ± 9.97	65.21 ± 9.75^*^	0.15	0.06	0.863	0.94
BF%	22.32 ± 4.43	22.14 ± 4.67	22.29 ± 4.91	21.04 ± 4.79^**^	22.68 ± 7.02	22.1 ± 7.3^*^	0.18	0.14	0.982	0.868
SLM	45.66 ± 8.3	45.9 ± 8.05	48.24 ± 7.77	48.19 ± 7.57	47.78 ± 7.4	48.05 ± 6.99	0.37	0.35	0.693	0.709
YBTs	1.84 ± 0.19	1.84 ± 0.17	1.81 ± 0.18	1.83 ± 0.17^*^	1.71 ± 0.23	1.74 ± 0.23^**^	1.31	0.97	0.283	0.388
HR	72.33 ± 8.64	63.75 ± 7.16^**^	74.92 ± 12.46	68.83 ± 7.578	72.75 ± 11.28	68.33 ± 9.44	0.19	1.43	0.825	0.254
SBP	110.83 ± 9.96	116.5 ± 8.55^*^	116.92 ± 9.28	109.67 ± 9.98^*^	114.25 ± 13.87	106.5 ± 9.95^*^	0.84	2.0	0.422	0.043
DBP	62.25 ± 7.59	59.0 ± 8.68	62.92 ± 15.33	66.58 ± 6.67	60.25 ± 9.23	65.17 ± 11.7	0.18	2.28	0.833	0.118
PP	51.833 ± 3.66	54.25 ± 2.73^*^	50.83 ± 3.95	49.0 ± 4.67	51.25 ± 6.88	48.25 ± 5.79	0.12	6.13	0.89	0.005

BF%, Body fat percentage; SLM, Soft Lean Mass; HIIT, High-intensity interval training; MICT, Moderate-intensity continuous training; HR, Heart rate; PP, Pulse pressure; YBTs, Y-Balance Tests; SBP, Systolic Blood Pressure; DBP, Diastolic Blood Pressure; PP, Pulse pressure. ^*^p < 0.05, ^**^p < 0.01.

### 3.2 Changes in arterial stiffness before and after training

**Figure 3:**
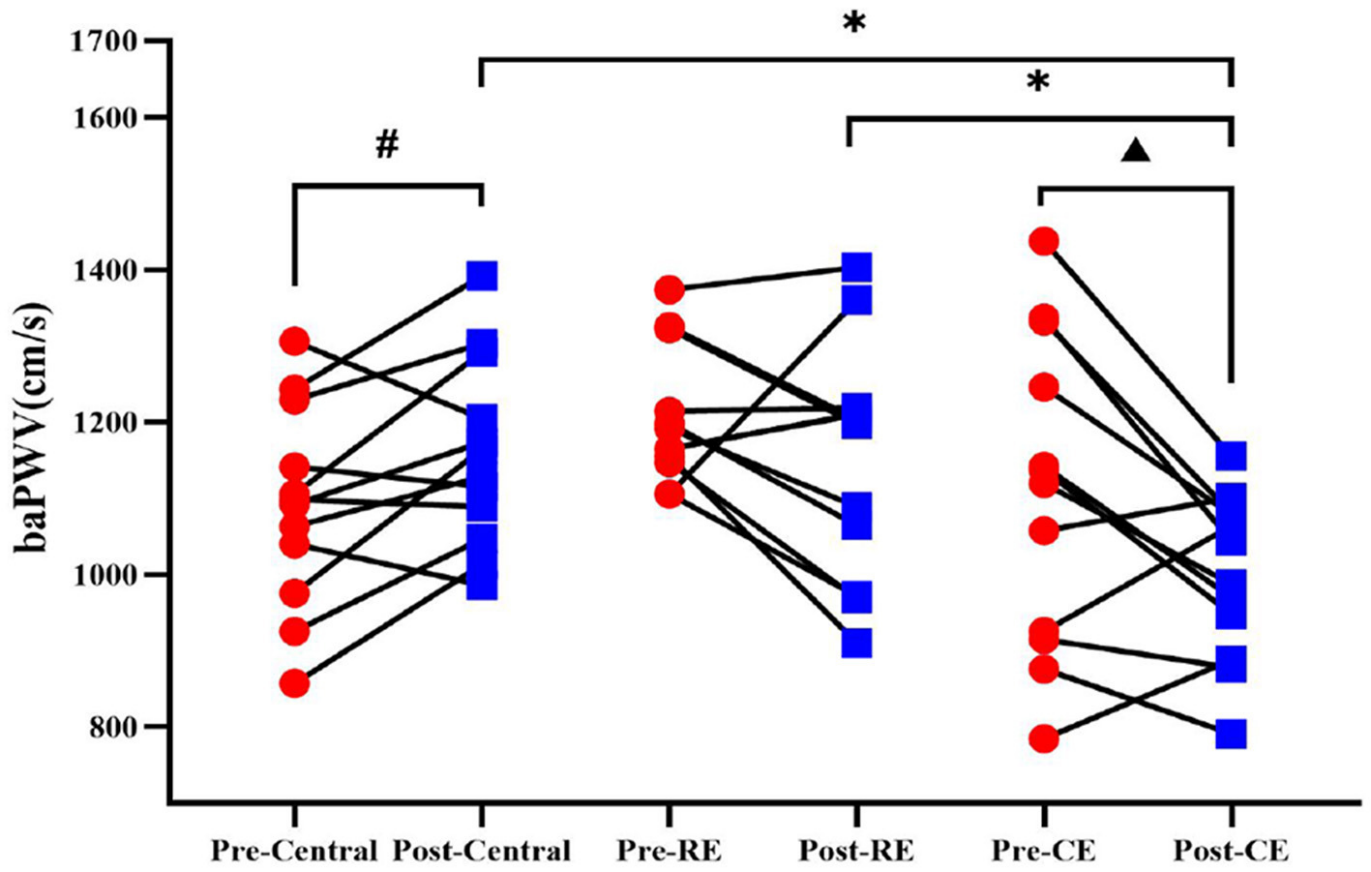
baPWV of participants before and after training. Comparison of the baPWV results between the pre-test and post-test measurements among the control group, room temperature group, and cold environment group. #Indicates that there was a significant difference between the control group before and after the test; ▴ indicates that there was a significant difference between the cold environment group before and after the test; *indicates that there was a difference between the groups (*P* < 0.05).

After training, the cold environment group showed significant decreases in baPWV (t = 2.626, *p* = 0.024), as shown in Figure 3 (*p* < 0.05). The average reduction of 11% in baPWV. In contrast, in the control group, the baPWV (t = −2.464, *p* = 0.31) increased significantly compared with those in the pretest group. In the room temperature group, baPWV did not change significantly. In the intergroup analysis, there was no difference in the pretest group, while there was a significant difference in baPWV (F = 5.845, *p* = 0.007) in the posttest group (*p* < 0.05). The comparison of their mean values showed that the baPWV of the cold environment group was lower than that of the room temperature group and the control group, and there was no significant difference between the room temperature group and the control group (*p* > 0.05).

### 3.3 Changes in oxygenated hemoglobin and total hemoglobin during the YBTs before and after training

**Figure 4:**
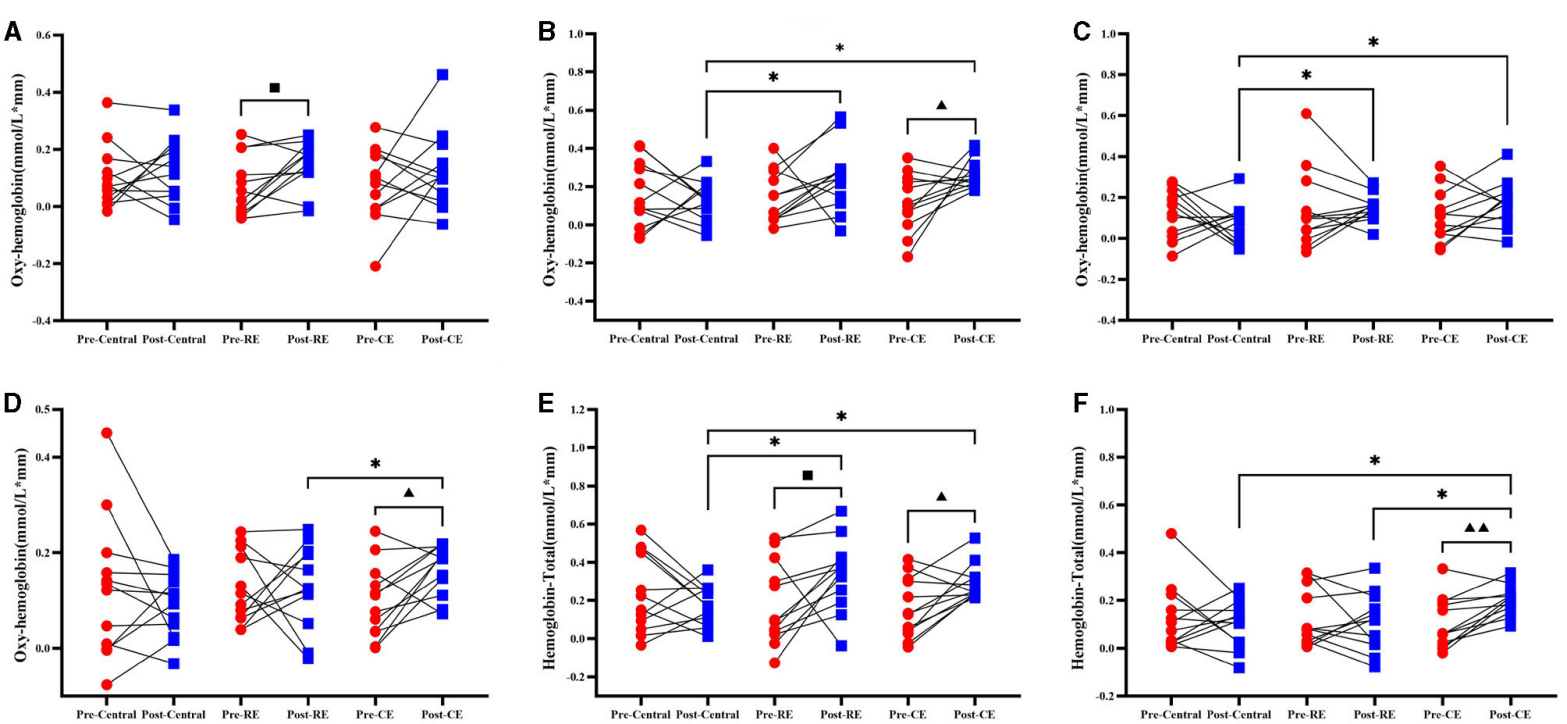
Oxygenated hemoglobin and total hemoglobin in participants before and after training when they were tested on the right side of the leg. Compares the results of oxygenated hemoglobin (HBO) and total hemoglobin (HBT) before and after the control group, room temperature group and cold environment group: **(A)** R–PMC and SMC; **(B)** L–M1; **(C)** R–S1; and **(D)** R–DLPFC. These 4 groups represent the changes in HBO and the comparison between groups. **(E)** L-M1; **(F)** R-DLPFC, which represent changes in HBT and comparisons between groups. ■ indicates that there is a significant difference between the room temperature group before and after the test (■, *P* < 0.05); ▴ indicates that there is a significant difference between the cold environment group before and after the test (▴, *P* < 0.05; ▴▴, *P* < 0.01); *Indicates that there is a difference between the groups (*, *P* < 0.05).

The HBO of the cold environment group was compared before and after training, and it was found that the concentration of oxygenated hemoglobin in the L-M1 (t = −2.543, *p* = 0.027), as shown in Figure 4B, and in the R-DLPFC (t = −2.775, *p* = 0.018), as shown in Figure 4D, increased significantly after training (*p* < 0.05). Figure 4A shows that the concentration of oxygenated hemoglobin of the room temperature group in the R-PMC and SMC (T = −2.641, *p* = 0.023) increased significantly before and after the test (*p* < 0.05), and the concentration of oxygenated hemoglobin in all brain regions in the control group did not change significantly (*p* > 0.05).

The intergroup comparison results of HBO showed that no intergroup differences were found in the pretest (*p* > 0.05), but on the posterior side, L-M1 (Figure 4B; F = 4.094, *p* = 0.026), R-S1 (Figure 4C; F = 4.027, *p* = 0.027), and R-DLPFC (Figure 4D; F = 3.352, *p* = 0.047) showed significant differences (*p* < 0.05). The changes in HBO concentrations in the cold environment group and the room temperature group in L-M1, as shown in Figure 4B, and R-S1, as shown in Figure 4C, were significantly greater than those in the control group. The HBO concentration in the cold environment group in the R-DLPFC was significantly greater than that in the control group (*p* < 0.05), and there was no significant difference between the cold environment group and the room temperature group (*p* > 0.05). The changes in the right HBO of the three groups of participants before and after training in all brain regions and the comparisons between groups are shown in Supplementary material 3.1.

Compared with those in the pretest, the concentrations of total hemoglobin in the cold environment group in the L-M1 (Figure 4E; t = −2.829, *p* = 0.016) and R-DLPFC (Figure 4F; t = −3.87, *p* = 0.003) brain regions were significantly greater after training (*p* < 0.05). The concentration of HBT in L-M1 (Figure 4E; t = −2.236, *p* = 0.047) in the room temperature group significantly increased (*p* < 0.05). There was no significant change in the concentration of total hemoglobin in any brain region in the control group (*p* > 0.05).

The intergroup comparison results of the HBT pretest also showed that there was no difference between the groups (*P* > 0.05), but there were significant differences in L-M1 (Figure 4E; F = 4.4487, *p* = 0.019) and R-DLPFC (Figure 4F; F = 3.613, *p* = 0.038) in the posttest (*p* < 0.05). As shown in Figure 4E, the HBT concentrations in the cold environment group and the room temperature group in L-M1 were significantly greater than those in the control group, and the HBT concentrations in the cold environment group, in the R-DLPFC were significantly greater than those in the room temperature group and the control group (*p* < 0.05). The changes in the right HBT of the three groups of participants before and after training in all brain regions and the intergroup comparisons are shown in Supplementary material 2.1.

### 3.4 Changes in oxygenated hemoglobin and total hemoglobin during the YBTs before and after training

**Figure 5:**
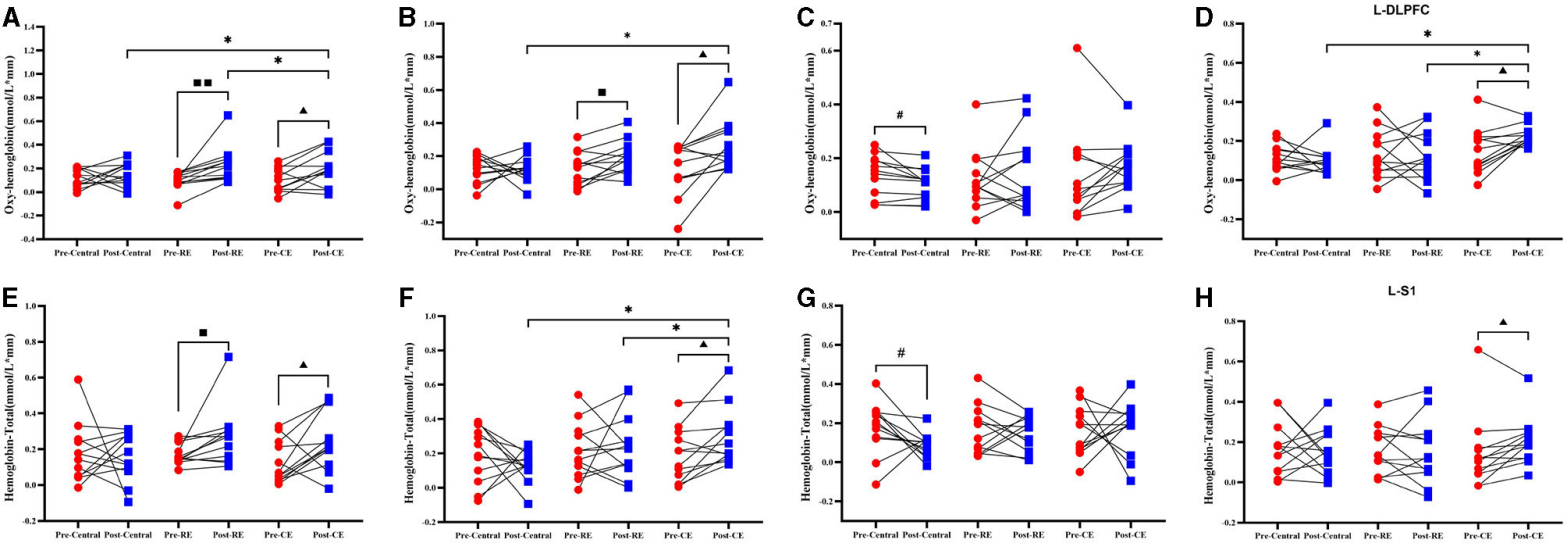
The oxygenated hemoglobin and total hemoglobin of the participants before and after training when they were tested in the left single-leg support test. Compares the results of oxygenated hemoglobin and total hemoglobin before and after the control group, room temperature group and cold environment group: **(A)** L-PM C and SMC; **(B)** R-M1; **(C)** L-S1; and **(D)** L-DLPFC. These four groups represent the changes in HBO and the comparison between groups. **(E)** L-PM C and SMC; **(F)** R-M1; **(G)** L-M1; and **(H)** L-S1, represents the changes in HBT and the comparison between groups. #Indicates that there is a significant difference between the control group before and after the test (#, *P* < 0.05); ■ indicates that there is a significant difference between the room temperature group before and after the test (■, *P* < 0.05; ■■, *P* < 0.01); ▴ indicates that there is a significant difference between the cold environment group before and after the test (▴, *P* < 0.05); *indicates that there is a difference between the groups (*, *P* < 0.05).

The HBO values of the cold environment group before and after training were compared, and the concentrations of oxygenated hemoglobin in the L-PMC and SMC brain regions (t = −2.747, *p* = 0.019), as shown in Figure 5A, the R-M1 (t = −2.989, *p* = 0.012), as shown in Figure 5B, and the L-DLPFC (t = −2.967, *p* = 0.013), as shown in Figure 5D; these values increased significantly after training (*P* < 0.05). The concentrations of oxygenated hemoglobin in the brain regions of the L-PMC and SMC (t = −3.348, *p* = 0.007) and R-M1 (*p* = −2.766, *p* = 0.018) were significantly greater than those in the pretest (*P* < 0.05). As shown in Figure 5C, in the control group, the concentration of HBO in L-S1 (*p* = 2.247, *p* = 0.046) before the pretest was significantly greater than that after the posttest (*P* < 0.05).

The intergroup comparison results of HBO showed that there was no difference between the groups in the pretest (*p* > 0.05), but in the posttest, R-M1 (F = 4.733, *P* = 0.016), as shown in Figure 5B and L-DLPFC(F = 7.442, *p* = 0.002), as shown in Figure 5D exhibited significant difference between the two groups (*p* < 0.05). As shown in Figure 5B, the HBO concentration in the cold environment group in R-M1 was significantly greater than that in the control group, but there was no significant difference from that in the RE group (*p* > 0.05). As shown in Figure 5D, the HBO concentration in the cold environment group in the L-DLPFC was significantly greater than that in the control group and the room temperature group (*p* < 0.05). The changes in the right HBO of the three groups of participants before and after training in all brain regions and the comparisons between the groups are shown in Supplementary material 3.2.

Compared with the HBT before the training, the cold environment group had a significant increased in L–PMC and SMC (Figure 5E; t = −2.374, *p* = 0.037), R-M1 (Figure 5F; t = −2.877, *p* = 0.015), and L-S1 (Figure 5H; t = −2.265, *p* = 0.045) after training. The concentration of total hemoglobin in the brain regions of the room temperature group significantly increased (*p* < 0.05), as shown in Figure 5E, L-PMC and SMC (t = −2.286, *p* = 0.043). The concentration of HBT in the brain regions of the control group significantly increased (*p* < 0.05). In Figure 5G of the control group, the concentration of L-M1 (t = 2.215, *p* = 0.049) total hemoglobin in the brain region was significantly decreased (*p* < 0.05).

The intergroup comparison results of HBT showed that there was no difference between the groups in the pretest (*P* > 0.05). According to the intergroup comparison of the posttest data, R-M1 (F = 3.765, *p* = 0.034) in Figure 5F had a significant difference (*p* < 0.05), and the concentration of HBT in the cold environment group was significantly higher than that in the room temperature group and the control group (*p* < 0.05). The one-way ANOVA results for L-M1 (F = 2.577, *p* = 0.091) in Figure 5F, indicate no significant differences among the three groups. However, significant difference was found between the cold environment group and the control group, indicating that the cold environment group can effectively improve cerebral hemodynamics. The changes in the right HBT of the three groups of participants before and after training in all brain regions and the comparisons between groups are shown in Supplementary material 2.2.

## 4 Discussion

This study investigated the effects of 1 week of high-intensity interval training in a cold environment on AS and cerebral hemodynamics in sedentary Chinese female college students after COVID-19. The main findings of this study are as follows: (1) The weight and body fat percentage of participants in the cold environment group decreased significantly, while the body fat percentage of the room temperature group decreased significantly. (2) The baPWV and PP of the cold environment group decreased significantly, while the systolic blood pressure of the control group increased significantly. Compared with that of the other groups, the AS of the cold environment group significantly improved, while the AS of the control group significantly increased. (3) The YBTs scores of the cold environment group and the room temperature group increased significantly. (4) The brain activation intensity and hemodynamics improved significantly in the cold environment group and the room temperature group, and the improvement in the cold environment group was significantly greater than that in the room temperature group. However, cerebral hemodynamics decreased in the control group.

Our results showed that the body fat percentage of both the room temperature group and the cold environment group decreased significantly, and the body fat percentage of the room temperature group decreased more than that of the cold environment group. This maybe because the main energy source for maintaining body temperature in a cold environment is glycogen. Previous studies have shown that at 21°C or 9°C, the oxidation utilization of carbohydrates increases, and the oxidation utilization rate of lipids decreases (43). The body weight of the cold environment group decreased significantly, this maybe because cold exposure caused a significant increase in energy consumption, while the decrease in ambient temperature caused a significant negative correlation with energy intake (44).

PP and baPWV are two major indicators of AS and are important predictors of cardiovascular risk and age-related decreases in the glomerular filtration rate (GFR) (45). baPWV was significantly correlated with aortic pulse wave velocity (46). In this study, the cold environment group had a significantly greater effect on improving AS in sedentary female college students than did the room temperature group, while AS increased significantly in the control group. The systolic blood pressure of the cold environment group and the room temperature group decreased significantly, while the systolic blood pressure of the control group increased significantly, and the heart rate of the control group decreased significantly. During HIIT, an increase in shear force leads to an increase in endothelial cell NO production, which promotes vascular smooth muscle relaxation and reduces systolic blood pressure and AS (47, 48). The cyclic reaction of muscle shivering heat production and adipose tissue metabolic activation during cold exposure may lead to an increase in ROS. As the body adapts to cold environments, it can enhance the adaptation of the body's antioxidant system, reduce the interference of reactive oxygen species in the body, and increase the bioavailability of NO (49). By adding cold stimulation to the exercise environment, changes in arterial diameter, greater blood flow and shear force can be mediated (33). CE exercise can induce a decrease in pulse pressure, increase aortic lumen diameter and wall thickness, stimulate angiogenesis and arteriole formation, increase collagen and elastin content, thereby improving AS (50), and promote the adaptation of the neuro-immune-endocrine network, such as the secretion of norepinephrine, and the secretion of a series of factors that promote cardiovascular health, including irisin and FGF21, which can promote arterial elasticity.

Sensory systems, muscle activation, and passive dynamics (i.e., ligaments and joints) must be coordinated with the central nervous system to achieve balance and control (51). While performing the challenging YBTs dynamic balance task, the main activated frontal cortex areas include the dorsolateral prefrontal cortex (DLPFC), premotor cortex (PMC), supplementary motor area (SMC), orbitofrontal cortex (OFC), frontopolar area (FPA), inferior frontal gyrus (IFG), primary motor cortex (M1), and primary sensory cortex (S1) (52–55). M1 and S1 are important structures in motor control. It receives and processes inputs from almost all cortical areas involved in motor control, including PMC and SMC, and sends motor commands through the corticospinal tract to regulate postural control. Greater activation of M1 may be associated with improved balance in single-leg standing (56, 57). The SMC and PMC are part of the indirect motor pathway, which mainly plans and prepares for movements and is activated in complex motor tasks or complex general tasks (58–60). The DLPFC is involved in allocating attention resources to maintain postural control and integrating external information with body position information (61). The activation of the DLPFC indicating that participants needed more cognitive resources to plan and regulate movements during the task.

In the comparison of left and right brain area activation, it was found that the left brain area with significantly increased activation before and after training was more than the right brain area. Studies have shown that there is clear asymmetry between the dominant leg and the non-dominant leg during a single-leg standing dynamic balance task (62). The brain resources required during the support movement of the non-dominant leg require more brain areas to be activated (63). The YBTs showed that there was no significant change in the control group, while the scores in the cold environment group and the room temperature group increased significantly, indicating that the dynamic balance ability of the participants significantly improved after HIIT. The score of the CE group was significantly greater than that of the RE group, mainly because in the cold environment, to maintain the temperature, the muscles provide the body with sufficient heat and increase the amount of muscle recruitment (64), thereby improving the motor function and work efficiency of the muscles.

This experiment revealed that cold-environment stimulation combined with exercise significantly increased cerebral hemodynamics in CE group. The cerebral hemodynamics of RE group was significantly improved, and the improvement amplitude was smaller than that of CE group. In the control group, the oxygenated hemoglobin concentration of L-S1 and the total hemoglobin concentration of L-M1 decreased significantly during the YBTs in the left single-leg support position. One study revealed that after sitting for 8 h, the CBF velocity decreased by approximately 10 cm/s compared with that at baseline (65). There was a negative correlation between sitting time and regional CBF in the frontal lobe (66). At the same time, the cardiovascular disease risks caused by SB (6), such as increased blood pressure, insulin resistance and dyslipidemia, have a negative impact on white matter (WM) health, thereby reducing brain activation intensity.

Regular exercise can enhance the production of angiogenic factors, induce angiogenesis and neurogenesis in the brain, increase cerebral perfusion and improve metabolism. Changes in brain hemodynamics are related to exercise intensity, and the blood flow velocity of the middle cerebral artery (MCAv) increases linearly with increasing exercise intensity. Cold environmental stimulation causes sympathetic nerve excitement, which causes the contraction of peripheral blood vessels in the skin to limit heat loss and maintain core temperature (67), resulting in increased blood flow in the aorta and increased cerebral blood flow perfusion. The cerebrovascular system is different from the peripheral microvascular system. Cold stimulation reduces cerebral vascular resistance and increases cerebral blood flow velocity (32, 33). At the same time, the dynamic cerebrovascular autoregulatory mechanism (dCA) is affected by sympathetic nerve activity. The dynamic relationship between blood pressure and middle cerebral artery blood flow velocity can be regulated when cold environmental stimulation causes changes in cerebral perfusion pressure (CPP). It protects fragile cerebral microvessels while maintaining sufficient blood flow to the brain and prevents excessive perfusion, which is crucial for preventing cerebral ischemia or cerebral hemorrhage (68).

Studies have shown that exercise in a cold environment increases the function of the glycolytic system, causing a significant increase in the concentration of lactate in the body (69). At the same time, the reduction in blood flow caused by vasoconstriction may lead to a delay (70), which will lead to a further increase in lactate accumulation in muscles. HIIT can effectively release lactate from muscles during intervals, providing sufficient energy for neuronal activity while also promoting neuronal activation and excitation, thereby promoting brain plasticity and improving cognitive ability (71). Therefore, we speculate that in addition to the increase in cerebral blood flow being an important factor in enhancing the functional activation of brain regions, the increase in substances such as lactate, irisin, FGF21, and myristic acid in a cold environment and the effective release of lactate and stimulation of neurons in HIIT can effectively promote the improvement of brain plasticity, which is an important influencing factor for the significant increase in brain activation in the cold environment group compared to the room temperature group during the YBTs.

Increased large artery stiffness can negatively affect brain health, resulting in cerebral blood flow disorders, reduced total brain volume (72), white matter hyperdensity (73) and increased cortical thickness (74). It can also lead to increased cerebral AS (75). The key mechanism by which greater AS leads to cerebral arterial dysfunction in mice may be the production of superoxide, which inhibits the bioavailability of nitric oxide and increases neuroinflammation (76–78). It was also found that there was a positive correlation between cerebral AS and cerebral perfusion; that is, greater cerebral AS manifested as reduced cerebral blood flow (79). Large artery stiffness can lead to increased PP, which will exert circumferential stress on the vascular wall, increase the risk of damage to cerebral arterioles and microvessels, and lead to impaired self-regulation of cerebral arteries, such as hypoperfusion imaging in the frontal and parietal white matter and hippocampus of adults (80). This finding is similar to our results. In the cold environment group, AS was significantly reduced, while blood flow and activation in most brain regions were significantly increased. In the control group, AS was significantly increased, while blood flow in some brain regions was significantly reduced.

## 5 Conclusion

High-intensity interval training in a cold environment has a significant effect on improving body fat percentage, AS, and cerebral hemodynamics in sedentary female college students after COVID-19. This shows that a cold environment, as a supplement to exercise, can promote cardiovascular health and brain function through multiple mechanisms, including increasing vascular shear force, stimulating the antioxidant system, and increasing cerebral blood flow. This provides a scientific basis for combining cold stimulation with high-intensity interval training to prevent cardiovascular disease and promote brain health. Future studies can further explore the effects of exercise in a cold environment on different populations and further analyze its mechanism of action.

## 6 Limitations

The sample size of this study was relatively small and included only sedentary Chinese female college students after COVID-19; therefore, the findings may not be generalizable to other populations, such as men, individuals in other age groups, or individuals with different physical constitutions. One week of high-intensity interval training in a cold environment was the only short-term intervention. Long-term effects and different intervention durations may lead to different results. There is a lack of cold stimulation with multiple temperature gradients, which can determine the most suitable cold stimulation temperature range.

## Data Availability

The data analyzed in this study is subject to the following licenses/restrictions: the data that support the findings of this study are available on request from the corresponding author. The data are not publicly available due to privacy or ethical restrictions. Requests to access these datasets should be directed to 18846777635@163.com.
