# Supplementary material for: Effects of high-intensity interval training in a cold environment on arterial stiffness and cerebral hemodynamics in sedentary Chinese college female students post-COVID-19
Source: Front Neurol. 2024 Nov 5;15:1466549. doi: 10.3389/fneur.2024.1466549 (PMC11573531; doi:10.3389/fneur.2024.1466549)
Supplement: Supplementary file 1 [file Table_1.DOCX]

**Appendix A**

**Appendix A.1**

**1.Analysis method of functional near-infrared spectroscopy**

The raw data were preprocessed using the HOMER2 toolbox (version 2.8) included with MATLAB R2013b (MathWorks, Natick, MA, USA). The preprocessing steps were as follows: the raw NIRS light intensity was converted to optical density signals; motion artifacts were detected by channel using the HOMER2 built-in function (parameters were set as tMotion = 1 s; tMAsk = 2.0; STDEVthresh = 15.0; AMPthresh = 5.0); detected motion artifacts were corrected by the spline interpolation method (hmrMotionCorrectSpline); a bandpass filter (0.01–0.1 Hz) was used to remove most of the systemic hemodynamic components, including those from the cardiac cycle (approximately 1 Hz) and respiration (approximately 0.2–0.3 Hz); and the modified Beer–Lambert law was applied to convert the filtered optical density data into oxyhemoglobin and deoxyhemoglobin. For each preprocessed experimental dataset, generalized linear models (GLMs) were used to analyze the HbO and HBT time series data. The mean HbO and HBT values were calculated for each ROI.

**2. Changes in total hemoglobin (HBT) before and after training**

**2.1 Changes in the right HBT scores of the three groups of participants before and after training and comparisons between the groups (Mean ± SD)**

|  | **Control(n=12)** | |  | **MICT(n=12)** | |  | **HIIT(n=12)** | |  | **F** | |  | **P-value** | |
| --- | --- | --- | --- | --- | --- | --- | --- | --- | --- | --- | --- | --- | --- | --- |
|  | **Pre** | **Post** |  | **Pre** | **Post** |  | **Pre** | **Post** |  | **Pre** | **Post** |  | **Pre** | **Post** |
| R-PMC and SMC | 0.14±0.12 | 0.13±0.11 |  | 0.15±0.16 | 0.15±0.10 |  | 0.12±0.11 | 0.15±0.07 |  | 0.182 | 0.120 |  | 0.835 | 0.888 |
| L-PMC and SMC | 0.18±0.21 | 0.17±0.09 |  | 0.16±0.25 | 0.20±0.16 |  | 0.16±0.15 | 0.15±0.09 |  | 0.036 | 0.436 |  | 0.965 | 0.650 |
| R-M1 | 0.23±0.20 | 0.17±0.19 |  | 0.24±0.27 | 0.26±0.16 |  | 0.26±0.18 | 0.22±0.11 |  | 0.053 | 0.933 |  | 0.948 | 0.404 |
| L-M1 | 0.24±0.21 | 0.18±0.10 |  | 0.18±0.22 | 0.34±0.19* |  | 0.16±0.16 | 0.29±0.09* |  | 0.482 | 4.487 |  | 0.622 | 0.019* |
| R-S1 | 0.17±0.13 | 0.12±0.11 |  | 0.12±0.20 | 0.18±0.17 |  | 0.14±0.15 | 0.18±0.13 |  | 0.269 | 0.624 |  | 0.766 | 0.542 |
| L-S1 | 0.15±0.14 | 0.14±0.10 |  | 0.13±0.17 | 0.22±0.20 |  | 0.12±0.10 | 0.19±0.11 |  | 0.239 | 0.856 |  | 0.789 | 0.434 |
| R-DLPFC | 0.13±0.14 | 0.10±0.10 |  | 0.13±0.12 | 0.11±0.12 |  | 0.11±0.11 | 0.20±0.06* |  | 0.113 | 3.613 |  | 0.893 | 0.038* |
| L-DLPFC | 0.12±0.12 | 0.12±0.09 |  | 0.09±0.16 | 0.13±0.11 |  | 0.10±0.09 | 0.14±0.05 |  | 0.155 | 0.119 |  | 0.857 | 0.889 |

R-PMC and SMC (right, supplementary motor cortex and premotor cortex), L-PMC and SMC (left, supplementary motor cortex and premotor cortex), R-M1 (right, primary motor cortex), L-M1 (left, primary motor cortex), R-S1 (right, primary somatosensory cortex), L-S1 (left, primary somatosensory cortex), R-DLPFC (right, dorsolateral prefrontal cortex), and L-DLPFC (left, dorsolateral prefrontal cortex). * p<0.05, ** p<0.01

**2.2 Changes in the left HBT of participants before and after training and comparisons between groups (Mean ± SD)**

|  | **Control(n=12)** | |  | **MICT(n=12)** | |  | **HIIT(n=12)** | |  | **F** | |  | **P-value** | |
| --- | --- | --- | --- | --- | --- | --- | --- | --- | --- | --- | --- | --- | --- | --- |
|  | **Pre** | **Post** |  | **Pre** | **Post** |  | **Pre** | **Post** |  | **Pre** | **Post** |  | **Pre** | **Post** |
| RPMC and SMC | 0.14±0.11 | 0.13±0.07 |  | 0.16±0.08 | 0.16±0.23 |  | 0.14±0.14 | 0.16±0.08 |  | 0.092 | 0.153 |  | 0.912 | 0.859 |
| LPMC and SMC | 0.18±0.17 | 0.16±0.13 |  | 0.17±0.06 | 0.27±0.16* |  | 0.12±0.12 | 0.24±0.16* |  | 0.697 | 1.708 |  | 0.505 | 0.197 |
| RM1 | 0.20±0.16 | 0.13±0.09 |  | 0.22±0.16 | 0.24±0.19 |  | 0.19±0.15 | 0.30±0.16* |  | 0.098 | 3.765 |  | 0.907 | 0.034* |
| LM1 | 0.18±0.13 | 0.09±0.06* |  | 0.17±0.12 | 0.15±0.09 |  | 0.16±0.13 | 0.18±0.14 |  | 0.068 | 2.577 |  | 0.935 | 0.091 |
| RS1 | 0.17±0.16 | 0.15±0.10 |  | 0.17±0.16 | 0.16±0.21 |  | 0.14±0.18 | 0.18±0.10 |  | 0.123 | 0.174 |  | 0.885 | 0.841 |
| LS1 | 0.17±0.13 | 0.16±0.11 |  | 0.16±0.12 | 0.15±0.16 |  | 0.14±0.18 | 0.20±0.12* |  | 0.088 | 0.547 |  | 0.916 | 0.584 |
| RDLPFC | 0.18±0.18 | 0.12±0.12 |  | 0.15±0.10 | 0.16±0.14 |  | 0.14±0.18 | 0.19±0.12 |  | 0.175 | 0.798 |  | 0.840 | 0.459 |
| LDLPFC | 0.12±0.11 | 0.11±0.08 |  | 0.15±0.12 | 0.15±0.16 |  | 0.15±0.12 | 0.19±0.13 |  | 0.263 | 1.002 |  | 0.771 | 0.378 |

R-PMC and SMC (right, supplementary motor cortex and premotor cortex), L-PMC and SMC (left, supplementary motor cortex and premotor cortex), R-M1 (right, primary motor cortex), L-M1 (left, primary motor cortex), R-S1 (right, primary somatosensory cortex), L-S1 (left, primary somatosensory cortex), R-DLPFC (right, dorsolateral prefrontal cortex), and L-DLPFC (left, dorsolateral prefrontal cortex). * p<0.05, ** p<0.01

**3.Changes in oxygenated hemoglobin (HBO) before and after training**

**3.1 Changes in the right HBO of participants before and after training and comparisons between groups (Mean ± SD)**

|  | **Control(n=12)** | |  | **MICT(n=12)** | |  | **HIIT(n=12)** | |  | **F** | |  | **P-value** | |
| --- | --- | --- | --- | --- | --- | --- | --- | --- | --- | --- | --- | --- | --- | --- |
|  | **Pre** | **Post** |  | **Pre** | **Post** |  | **Pre** | **Post** |  | **Pre** | **Post** |  | **Pre** | **Post** |
| RPMC and SMC | 0.1±0.11 | 0.12±0.11 |  | 0.07±0.10 | 0.15±0.08* |  | 0.08±0.13 | 0.13±0.14 |  | 0.280 | 0.028 |  | 0.757 | 0.973 |
| LPMC and SMC | 0.09±0.12 | 0.13±0.10 |  | 0.13±0.18 | 0.13±0.13 |  | 0.11±0.12 | 0.12±0.10 |  | 0.292 | 0.021 |  | 0.749 | 0.979 |
| RM1 | 0.16±0.10 | 0.15±0.11 |  | 0.19±0.18 | 0.15±0.19 |  | 0.15±0.13 | 0.17±0.11 |  | 0.174 | 0.142 |  | 0.841 | 0.868 |
| LM1 | 0.16±0.17 | 0.13±0.11 |  | 0.14±0.14 | 0.24±0.17 |  | 0.12±0.15 | 0.26±0.08* |  | 0.205 | 4.094 |  | 0.816 | 0.026* |
| RS1 | 0.12±0.12 | 0.07±0.10 |  | 0.14±0.19 | 0.15±0.06 |  | 0.11±0.13 | 0.17±0.11 |  | 0.139 | 4.027 |  | 0.870 | 0.027* |
| LS1 | 0.15±0.15 | 0.13±0.09 |  | 0.13±0.12 | 0.21±0.20 |  | 0.12±0.09 | 0.18±0.07 |  | 0.241 | 1.029 |  | 0.787 | 0.368 |
| RDLPFC | 0.12±0.15 | 0.09±0.07 |  | 0.12±0.07 | 0.13±0.09 |  | 0.10±0.08 | 0.17±0.05* |  | 0.243 | 3.352 |  | 0.785 | 0.047* |
| LDLPFC | 0.10±0.11 | 0.12±0.05 |  | 0.11±0.12 | 0.12±0.10 |  | 0.11±0.08 | 0.13±0.05 |  | 0.036 | 0.059 |  | 0.964 | 0.943 |

R-PMC and SMC (right, supplementary motor cortex and premotor cortex), L-PMC and SMC (left, supplementary motor cortex and premotor cortex), R-M1 (right, primary motor cortex), L-M1 (left, primary motor cortex), R-S1 (right, primary somatosensory cortex), L-S1 (left, primary somatosensory cortex), R-DLPFC (right, dorsolateral prefrontal cortex), and L-DLPFC (left, dorsolateral prefrontal cortex). * p<0.05, ** p<0.01

**3.2 Changes in the left HBO of participants before and after training and comparisons between groups (Mean ± SD)**

|  | Control(n=12) | |  | MICT(n=12) | |  | HIIT(n=12) | |  | F | |  | P-value | |
| --- | --- | --- | --- | --- | --- | --- | --- | --- | --- | --- | --- | --- | --- | --- |
|  | Pre | Post |  | Pre | Post |  | Pre | Post |  | Pre | Post |  | Pre | Post |
| RPMC and SMC | 0.09±0.10 | 0.10±0.09 |  | 0.11±0.08 | 0.15±0.17 |  | 0.11±0.10 | 0.15±0.09 |  | 0.218 | 0.595 |  | 0.805 | 0.557 |
| LPMC and SMC | 0.12±0.08 | 0.15±0.10 |  | 0.10±0.08 | 0.23±0.15* |  | 0.10±0.10 | 0.20±0.15* |  | 0.204 | 1.216 |  | 0.816 | 0.309 |
| RM1 | 0.12±0.08 | 0.12±0.07 |  | 0.13±0.10 | 0.19±0.10* |  | 0.13±0.16 | 0.26±0.15* |  | 0.004 | 4.733 |  | 0.996 | 0.016* |
| LM1 | 0.13±0.12 | 0.09±0.08 |  | 0.12±0.12 | 0.15±0.08 |  | 0.12±0.16 | 0.16±0.12 |  | 0.013 | 2.059 |  | 0.987 | 0.144 |
| RS1 | 0.16±0.13 | 0.15±0.13 |  | 0.14±0.17 | 0.14±0.19 |  | 0.13±0.17 | 0.18±0.08 |  | 0.068 | 0.259 |  | 0.934 | 0.773 |
| LS1 | 0.13±0.08 | 0.11±0.06* |  | 0.12±0.11 | 0.14±0.14 |  | 0.13±0.17 | 0.17±0.10 |  | 0.035 | 1.132 |  | 0.966 | 0.335 |
| RDLPFC | 0.14±0.12 | 0.10±0.13 |  | 0.15±0.10 | 0.15±0.10 |  | 0.12±0.13 | 0.17±0.08 |  | 0.168 | 1.299 |  | 0.846 | 0.306 |
| LDLPFC | 0.11±0.07 | 0.10±0.07 |  | 0.13±0.12 | 0.12±0.12 |  | 0.15±0.12 | 0.23±0.05* |  | 0.313 | 7.442 |  | 0.733 | 0.002* |

R-PMC and SMC (right, supplementary motor cortex and premotor cortex), L-PMC and SMC (left, supplementary motor cortex and premotor cortex), R-M1 (right, primary motor cortex), L-M1 (left, primary motor cortex), R-S1 (right, primary somatosensory cortex), L-S1 (left, primary somatosensory cortex), R-DLPFC (right, dorsolateral prefrontal cortex), and L-DLPFC (left, dorsolateral prefrontal cortex). * p<0.05, ** p<0.01
